# Implementation of Competency-Based Education for Quality Midwifery Programs in Africa: Protocol for a Scoping Review

**DOI:** 10.2196/47603

**Published:** 2023-12-04

**Authors:** Waleola Bukola Ige, Winnie Baphumelele Ngcobo, Opeyemi Afolabi

**Affiliations:** 1 School of Nursing and Public Health College of Health Sciences University of KwaZulu-Natal South Africa Nigeria; 2 School of Nursing and Public Health College of Health Sciences University of KwaZulu-Natal Duban South Africa; 3 Department of Nursing Science Adeleke University Ede Osun State Nigeria

**Keywords:** Africa, competency-based education, implementation, midwifery programme, protocol, scoping review, sustainability

## Abstract

**Background:**

Competency-based education (CBE) for midwifery programs is a system of academic instruction and evaluation that aims to achieve proficiency in midwifery student learning outcomes, which is based on students’ ability to demonstrate the knowledge, attitudes, self-perceptions, and skills of a predetermined set of educational activities in theory and practice. CBE focuses on ensuring that midwifery students can develop critical thinking abilities, values, and the clinical decision-making abilities needed for the delivery of safe care in future practice.

**Objective:**

The objective of this scoping review is to map and synthesize existing literature on the implementation of CBE for midwifery programs and its sustainability in Africa.

**Methods:**

We will use the Arksey and O’Malley approach for scoping reviews for the research methodology. The 3-stage search process, proposed by the Joanna Briggs Institute, will be used to determine the eligibility of published and unpublished studies. PubMed, Science Direct, Web of Science, CINAHL, PsycINFO, and Scopus will be searched to screen published articles. ProQuest Dissertations and Theses and Google Scholar will be used to search for unpublished studies. Findings will only apply to studies conducted in Africa from 2010 to the present year in English. The 2 reviewers will work independently to carefully screen and compare the full text of the selected citations to the inclusion criteria. In the event of any disagreements between the 2 reviewers at any stage of the selection process regarding the inclusion of an article, this will be settled by discussion or consultation with a third reviewer. The extracted data will be presented using a PRISMA-ScR (Preferred Reporting Items for Systematic Reviews and Meta-Analyses Extension for Scoping Review) flow diagram with an attached narrative summary. The review will summarize and disseminate findings on the implementation of the CBE for midwifery programs and its sustainability in Africa.

**Results:**

It is intended that this scoping review will be completed within 6 months following the publication of this protocol.

**Conclusions:**

The conclusions from this scoping review will inform midwifery educators, institutions, policy makers, and other stakeholders on the strategies to implement and sustain CBE for midwifery programs in Africa.

**International Registered Report Identifier (IRRID):**

PRR1-10.2196/47603

## Introduction

The past few decades have witnessed a rapid and worldwide reform in traditional education toward competency-based education (CBE) at all levels and types of education [1,2]. The competency-based approach to education focuses on addressing positive educational outcomes that align with the needs and expectations of the workforce, employers, and the population they serve [3,4]. CBE places strong emphasis on learner-centered learning, which encourages better adaptability and accountability [5,6]. Through CBE, graduates can critically think, communicate effectively, and work collaboratively with others to provide solutions to problems [7]. This teaching and learning strategy differs from the traditional approach, which consists of time-based training, evidence of academic degrees, and the assessment of student progress based on lessons taught [8,9]. This paradigm shift evolved in an attempt to bridge the gap between students having completed their educational program yet still being unable to perform competently in the workplace or being inadequate to proceed to the next level of education [8,10], despite the immeasurable efforts, time, and resources invested in this traditional education without achieving positive outcomes [10].

The World Health Organization (WHO) and the International Confederation of Midwives (ICM) also adopted this educational trend in the education of midwives and other health professionals by using the term “core competencies” [11,12]. Core competencies, also known as essential competencies, are a set standard for preparing the health workforce to be current and responsive to dramatic health advances geared to meeting clients’ expectations [13,14]. The midwifery set of competencies was created and published by the ICM in 2002 [15]. Since then, the competencies have undergone reviews and updates based on scientific evidence [16,17]. The updated competencies are arranged in a framework of 4 interconnected categories, including general competence, which applies to all aspects of a midwife’s practice, and competencies that are particular to care throughout prepregnancy, antenatal care, labor, delivery, and the postpartum period [18]. These competencies represent the requirement for preservice education in midwifery and its intended outcomes [17]. CBE for the midwifery profession is, therefore, a system of academic instruction and evaluation that aims to achieve proficiency in midwifery student learning outcomes based on students’ ability to demonstrate all the essential competencies for basic midwifery practice [18].

CBE aims at developing critical thinking abilities, values, and the clinical decision-making abilities needed by midwifery students for the delivery of safe care in future practice [10,14]. The design of CBE for teaching and learning activities should comply with standards by integrating knowledge, skills, values, and attitudes within each competency [19]. Midwife educators, midwifery regulatory bodies, midwifery institutions, and others involved in the development and implementation of preservice midwifery education are expected to adopt and adapt CBE to curricula [12,14,20]. However, reports on the integration of competency-based curricula and the approach to ensure sustainability are yet to be well documented, especially in Africa [14], whereas countries and institutions in which the CBE approach has been fully adopted have recorded positive outcomes in their education programs. Outcomes include, but are not limited to, producing competent and confident graduates and improved quality care for clients, contributing to improved family health [8,21-23]. Previous reviews on CBE in Africa focused on challenges regarding the implementation of CBE [24,25]. This review will, however, explore and synthesize existing literature on the implementation of CBE for midwifery programs and its sustainability in Africa.

**Figure 1:**
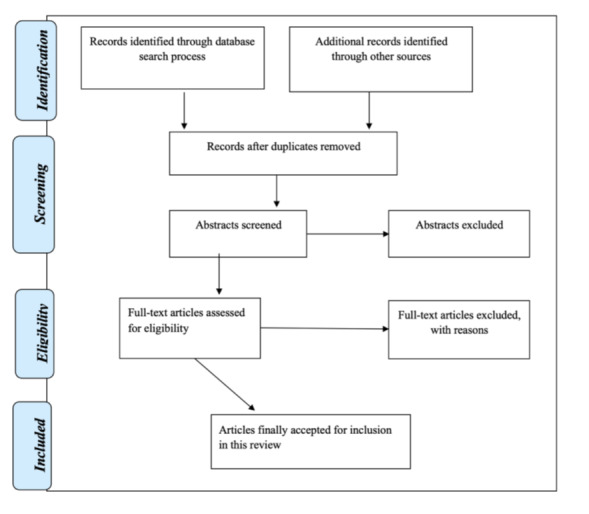
PRISMA-ScR (Preferred Reporting Items for Systematic Reviews and Meta-Analyses Extension for Scoping Review) flow diagram showing the phases of the literature search and selection procedures of studies for the review.

## Methods

### Overview

The Arksey and O’Malley [26] approach for scoping reviews will be used for the research methodology. The Arksey and O’Malley [26] framework comprises the following steps: (1) identify the research question; (2) identify relevant studies; (3) perform study selection; (4) extract and chart the data; and (5) collate, summarize, and report the results. This framework will direct the scoping review methodology, and, if applicable, the protocol will adhere to the pertinent PRISMA-ScR (Preferred Reporting Items for Systematic Reviews and Meta-Analyses Extension for Scoping Review) guidelines [27].

### Step 1: Identify the Research Question

The following research questions will guide this study:

1. What is the existing evidence on implementing CBE in midwifery education in Africa?

2. How has CBE for midwifery education been sustained in Africa?

#### Inclusion Criteria

The participants, concept, and context (PCC) framework will guide the inclusion of eligible studies in this scoping review [28].

#### Participants

Studies that include midwifery students, midwifery educators, midwives, obstetric nursing, obstetrics and gynecology nursing, and maternal and child health nursing relating to CBE will be included in this study, while studies on CBE relating to other health professions such as nursing, medicine, pharmacy, and allied health professions will be considered irrelevant to this study.

#### Concept

Studies that discuss the implementation of competency-based midwifery education in Africa will be considered in this scoping review. The concept of interest is competency-based curriculum and teaching and learning strategies used in theory and clinical practice. These strategies include the lecture method, demonstration, simulation, role play, etc. Competency-based curriculum content includes the ICM competency framework, that is, prepregnancy, pregnancy, labor and childbirth, and the postpartum period.

#### Context

The context covers academic settings, including, but not limited to, schools, colleges, polytechnics, universities, and hospitals facilitating the teaching and learning of midwifery education programs. These settings where CBE is integrated into theory and practice will be considered in this review. Studies that relate to Africa, published from 2010 onward, will be considered.

### Step 2: Identify Relevant Studies

The 3-stage search process proposed by Joanna Briggs Institute will be used to determine the eligibility of published and unpublished studies [29]. To develop a comprehensive search strategy, a preliminary limited search of PubMed and EBSCOhost will be performed to scrutinize text words found in the titles and abstracts, including any index keywords. Second, a second search across PubMed, EBSCOhost, Science Direct, and Scopus will be carried out using all specified keywords and index terms. Third, the reference lists of all accessed articles will be checked for additional studies and included, if considered eligible. Furthermore, ProQuest Dissertations and Theses and Google Scholar will be used to search for unpublished studies that would not have been easily accessed through conventional databases. Findings will only apply to studies conducted in Africa from 2010 to 2024, presented in English.

### Step 3: Perform Study Selection

The aggregate citations found will be uploaded into EndNote (version 9; Clarivate Analytics) after the search, while duplicates will be deleted manually. Titles and abstracts will then be screened by 2 independent reviewers (WBI and OA) to assess eligibility using the PCC framework. The full text of potentially relevant articles that fulfill the eligibility requirements will be retrieved, and their citation information will be imported into Rayyan (Qatar Computing Research Institute). WBI and OA will work independently to carefully screen and compare the full text of the selected citations to the inclusion criteria. The reasons for the exclusion of the full text of articles not considered will be documented and reported in the scoping review. In the event of any disagreements at any stage of the selection process regarding the inclusion of an article between WBI and OA, this will be settled by discussion or consultation with WBN (the third reviewer). The final scoping review will include a narrative report on the search and study selection procedure and a diagrammatic presentation using a PRISMA-ScR flow diagram [27].

### Step 4: Extract and Chart the Data

All eligible studies’ data will be extracted and processed using data charting. A data charting table will be created using Excel (Microsoft Corp) to extract and chart relevant information. The team of reviewers will pilot the data charting form on 5-10 articles to ensure that vital information is completely extracted. All publications meeting the inclusion criteria will have their data extracted by 2 independent reviewers, and the data will then be validated by a third reviewer. The reviewers will independently draft and later compare the form for consistency, using the PCC framework of the review objectives and questions, and may also consider including emerging relevant variables that may arise during the process. These variables currently consist of (but are not limited to) the type of study, year, setting or context, aims, study population, research design, research techniques, and summary of findings. If applicable, this draft will be modified and revised.

### Step 5: Collate, Summarize, and Report the Results

The aim of this study is to map existing evidence on the implementation of competency-based midwifery education across the various studies in Africa. The extracted data will be presented using a PRISMA-ScR flow diagram with an attached narrative summary. Descriptive statistical analysis (eg, frequencies) will initially be used to quantitatively summarize the extracted data. The reviewers will further analyze the data using a qualitative content analysis using the Graneheim and Lundman [30] approach to find new themes and present a narrative report and descriptive analysis of the studies included. This identified theme will be coded independently by the reviewers using NVivo software (version 12; QSR International) with reference to the variables mentioned earlier during the data extraction stage. To ensure validity and credibility, the team will discuss and cross-validate the findings, that is, the codes and emerging themes. The team will also address the implications for future research, clinical practice, and policy while critically evaluating the findings’ significance to the study’s primary objective.

## Results

It is intended that this scoping review will be completed within 6 months following the publication of this protocol. The findings will be presented as outlined above.

## Discussion

The purpose of this scoping review is to map and synthesize existing literature on the implementation of CBE for midwifery programs and its sustainability in Africa. The discussion of the findings will be contextualized based on previous studies on CBE for midwifery programs, its implementation, and the strategies used to sustain the implementation of CBE for midwifery programs in Africa. The scoping review is limited to midwifery education, which means other health care professionals’ education will be excluded. The review is also limited to studies conducted in Africa in the English language. The findings of the review will be significant in identifying gaps in knowledge and research on this topic of interest. It is anticipated that understanding CBE for midwifery programs may contribute to strengthening midwifery education and advancing the standard of maternity care. The conclusions from this scoping review will inform midwifery educators, institutions, policy makers, and other stakeholders on the strategies required to implement and sustain CBE for midwifery programs in Africa. The results of this scoping review will be published in a reputable journal and presented at local and international conferences.

## Abbreviations

CBE: competency-based education
ICM: International Confederation of Midwives
PCC: participants, concept, and context
PRISMA-ScR: Preferred Reporting Items for Systematic Reviews and Meta-Analyses Extension for Scoping Review
WHO: World Health Organization
